# Chronic Recurrent Multifocal Osteomyelitis Associated in a Child with Inflammatory Bowel Disease and Primary Sclerosing Cholangitis: A Case Report

**DOI:** 10.31662/jmaj.2025-0196

**Published:** 2025-09-19

**Authors:** Yuto Nishiura, Yuta Tsukagoshi, Daisuke Nozawa, Hirota Saito, Kenichi Honma, Toru Hoshi, Masazumi Suzuki, Yasuaki Hosono, Hayato Shimada, Shinsen Nomura, Yoshiyasu Ikezawa

**Affiliations:** 1 Department of Orthopaedic Surgery, Mito Saiseikai General Hospital, Ibaraki, Japan; 2 Department of Orthopaedic Surgery, Ibaraki Children’s Hospital, Ibaraki, Japan; 3 Department of Orthopaedic Surgery, Institute of Medicine, University of Tsukuba, Ibaraki, Japan; 4 Department of Pediatrics, Ibaraki Children’s Hospital, Ibaraki, Japan

**Keywords:** chronic recurrent multifocal osteomyelitis, inflammatory bowel disease, primary sclerosing cholangitis

## Abstract

Chronic recurrent multifocal osteomyelitis (CRMO) is a rare, non-infectious autoinflammatory bone disorder that predominantly affects children. Some studies have reported cases of CRMO in patients with autoimmune diseases such as inflammatory bowel disease (IBD). We encountered a rare pediatric case of CRMO in a patient with both IBD and primary sclerosing cholangitis (PSC). The patient was a 14-year-old male with a history of ulcerative colitis and PSC, both controlled with vedolizumab, who developed recurrent right wrist pain. Imaging revealed bone marrow edema suggestive of osteomyelitis. Initially suspected to be bacterial osteomyelitis, the patient was treated with antibiotics; however, symptoms recurred, and new lesions appeared on the heel, ultimately leading to a diagnosis of CRMO. The patient was successfully managed with analgesics alone, without antibiotics, and no long-term complications were observed. This is a rare pediatric case of CRMO occurring in the context of both IBD and PSC. Clinicians should consider CRMO in patients with IBD and PSC who present with musculoskeletal symptoms to avoid unnecessary antibiotic use. Early-stage diagnosis of CRMO can be achieved by detecting multifocal lesions on magnetic resonance imaging.

## Introduction

Chronic recurrent multifocal osteomyelitis (CRMO) is a rare and poorly understood non-infectious autoinflammatory bone disease characterized by recurrent episodes of bone pain and inflammation ^(1)^. It primarily affects children and adolescents, with a higher prevalence in females ^(2)^. CRMO commonly involves long bones, the spine, and the mandible ^(3)^, presenting as pain, swelling, and redness. The disease is frequently associated with autoimmune conditions such as inflammatory bowel disease (IBD), psoriasis, and palmoplantar pustulosis ^(4)^.

Primary sclerosing cholangitis (PSC) is a chronic cholestatic liver disease characterized by progressive inflammation and fibrosis of the bile ducts, often leading to liver cirrhosis. PSC is strongly associated with IBD, particularly ulcerative colitis (UC), and is considered an immune-mediated disorder. Although the exact relationship between CRMO and PSC remains unclear, both conditions involve dysregulated immune responses and are linked to IBD, suggesting a possible shared pathogenic mechanism ^(5)^.

The pathophysiology of CRMO remains unclear but likely involves innate immune dysregulation influenced by genetic and environmental factors. Unlike bacterial osteomyelitis, CRMO is sterile and unresponsive to antibiotics. Diagnosis relies on magnetic resonance imaging (MRI), which reveals bone marrow edema at multiple sites, with biopsy used to exclude infection or malignancy. In cases with concomitant PSC and IBD, immunological and genetic evaluations may provide further insights ^(6)^.

## Case Report

A 14-year-old male with UC, controlled with vedolizumab, presented with right wrist pain persisting for 2 months. He was diagnosed with UC and PSC at age 12 following persistent diarrhea, liver dysfunction, and biopsy.

**Figure 1.**
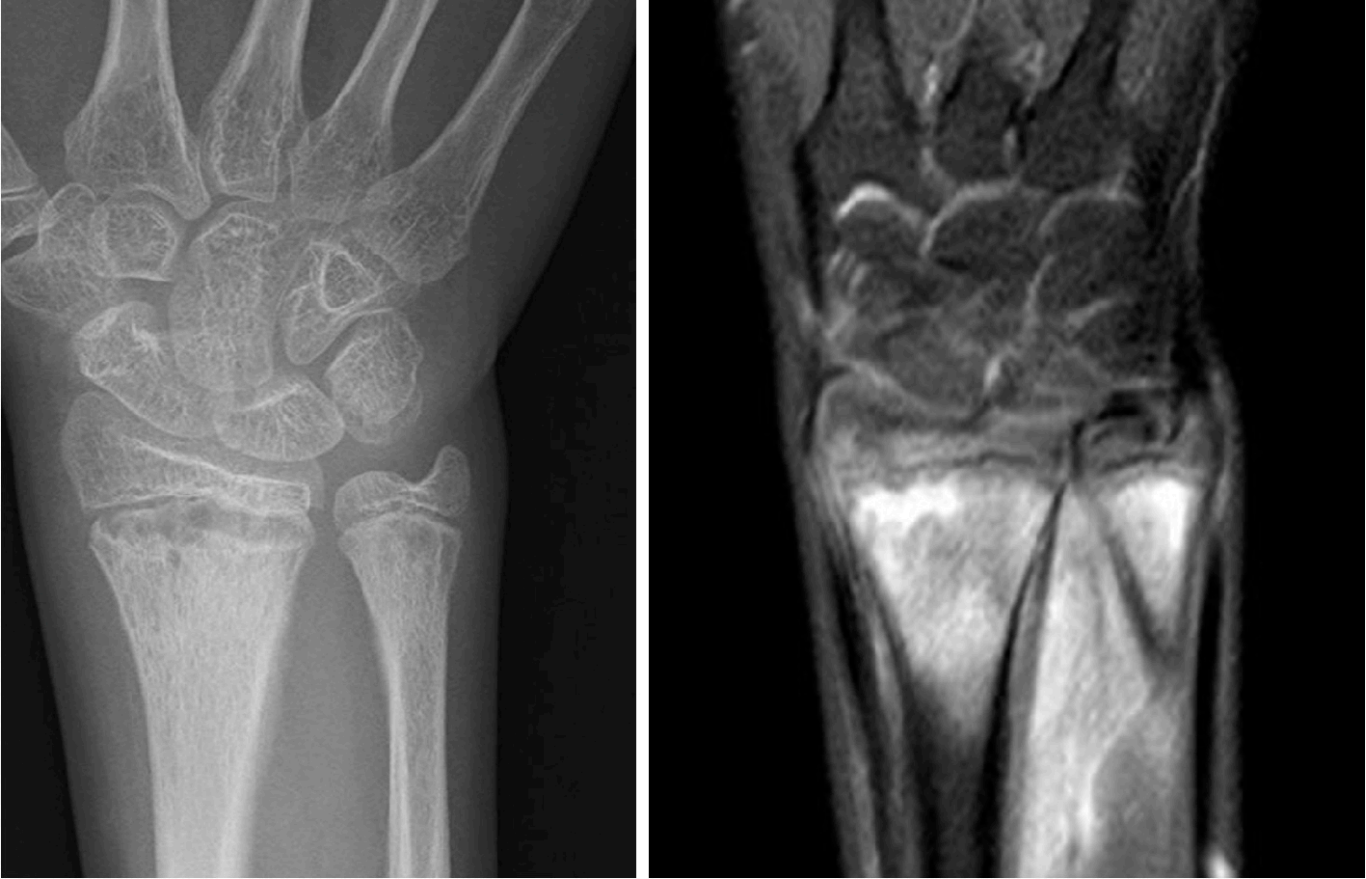
Radiograph and MRI of the heel performed on the initial visit. MRI with STIR sequences demonstrated bone marrow edema. MRI: magnetic resonance imaging; STIR: short tau inversion recovery.

Physical examination revealed redness and swelling of the right wrist, along with untreated gingivitis. Laboratory findings showed a white blood cell count of 11,700/μL and C-reactive protein 41.3 mg/L; however, blood cultures were negative. Ultrasound examination showed no synovitis, though a small amount of joint fluid was present; puncture fluid culture was also negative. Although MRI showed significant bone marrow edema, the absence of active synovitis on ultrasound made juvenile idiopathic arthritis unlikely. MRI (Short Tau Inversion Recovery) demonstrated bone marrow edema, suggesting osteomyelitis (Figure 1). Due to immunosuppression, hematogenous bacterial osteomyelitis was suspected, and empirical antibiotics were initiated. As C-reactive protein levels gradually decreased and symptoms resolved, antibiotics were discontinued.

**Figure 2.**
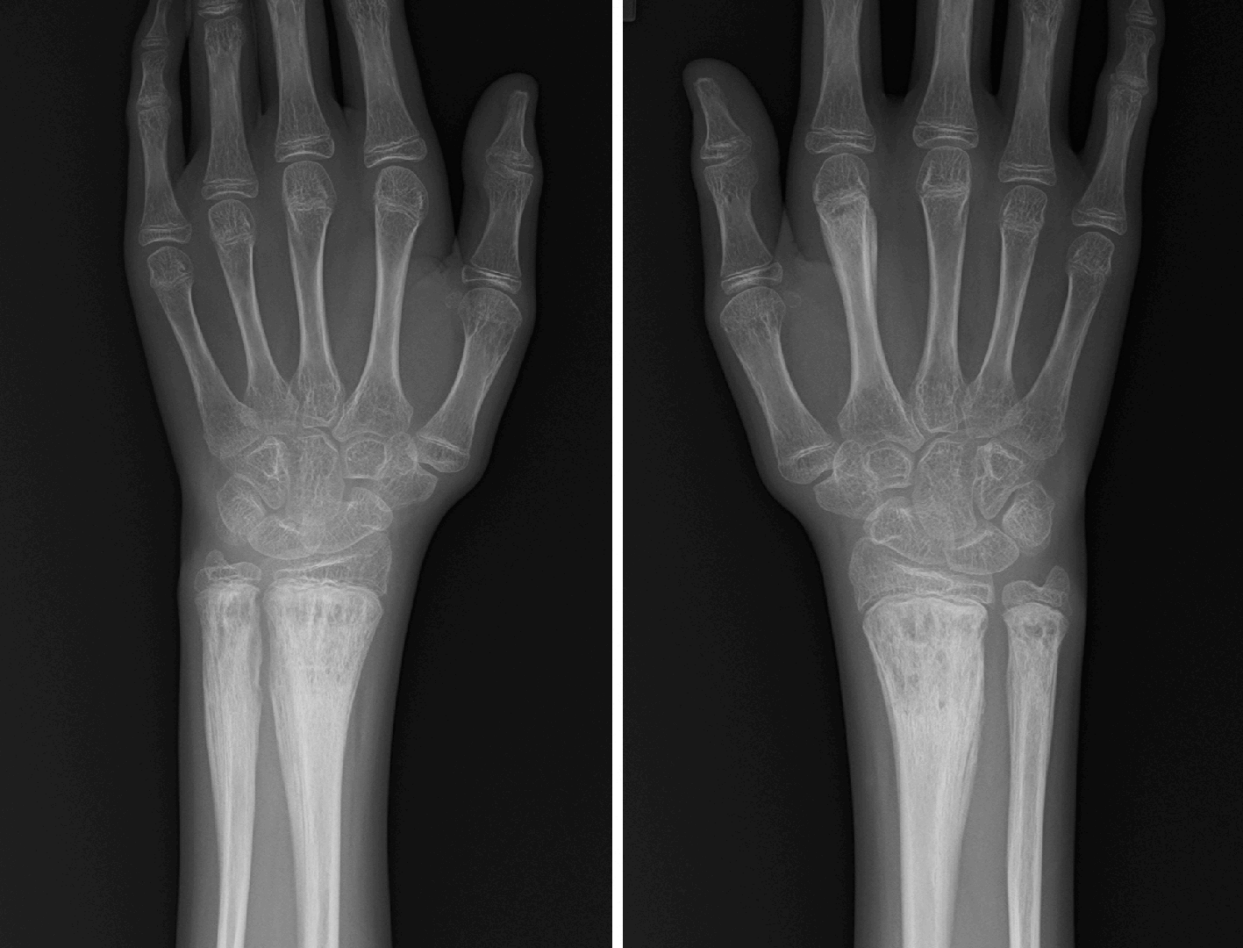
Radiograph of both wrists performed 6 months after the initial visit. Radiographs showing chronic osteomyelitis, though the contralateral wrist was asymptomatic.

**Figure 3.**
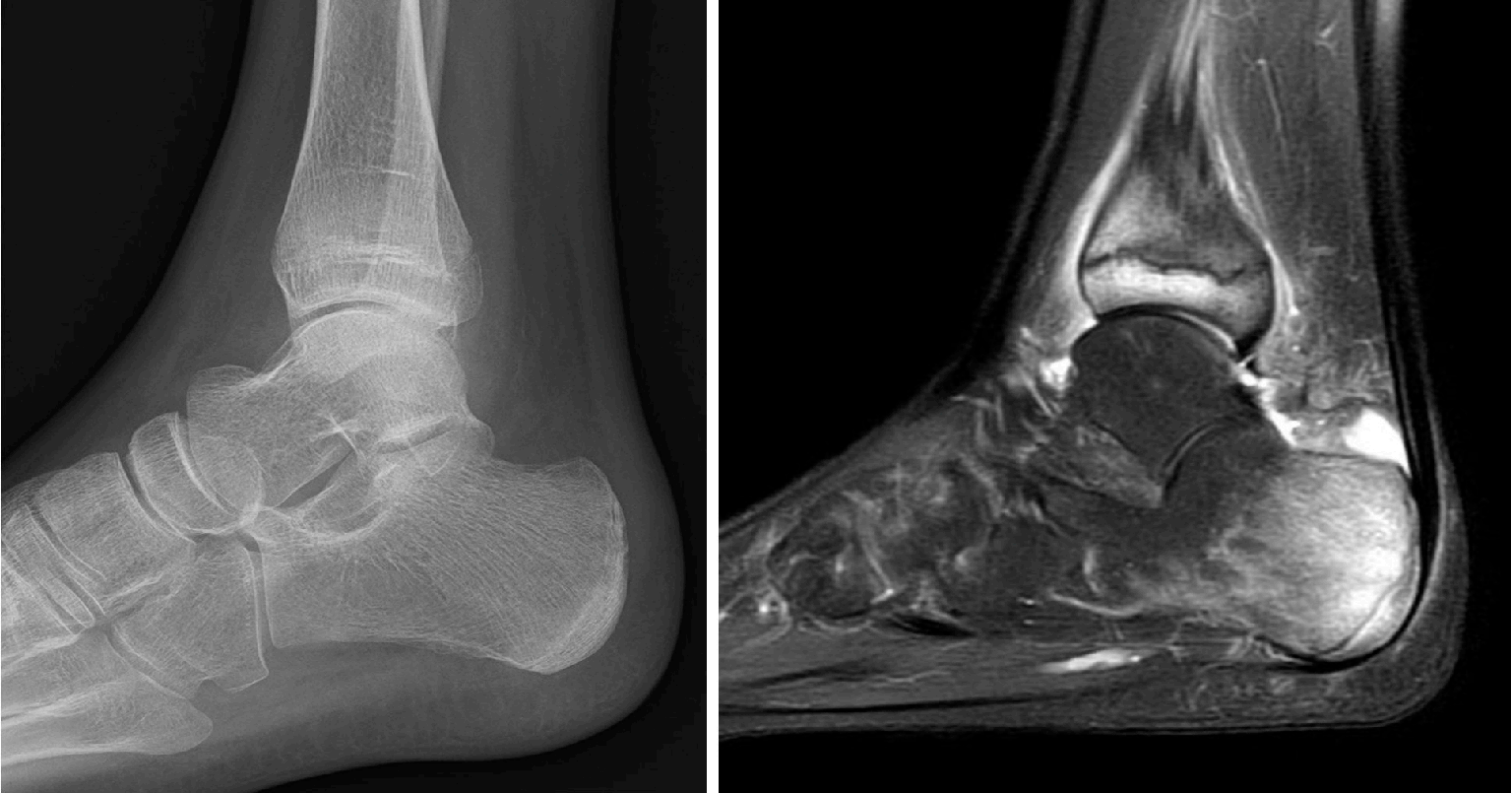
Radiograph and MRI of the heel performed 1 year and 6 months after the initial visit. MRI revealed bone marrow edema, despite no abnormalities being observed on plain radiography. MRI: magnetic resonance imaging

Six months later, wrist pain recurred. Radiographs of both wrists revealed chronic osteomyelitis, although the contralateral wrist remained asymptomatic (Figure 2). Given the patient’s immunosuppression state, a bacterial flare-up was suspected, and antibiotics were resumed. Symptoms again resolved. One year and 6 months later, the patient developed right heel pain, and MRI confirmed bone marrow edema (Figure 3).

**Figure 4.**
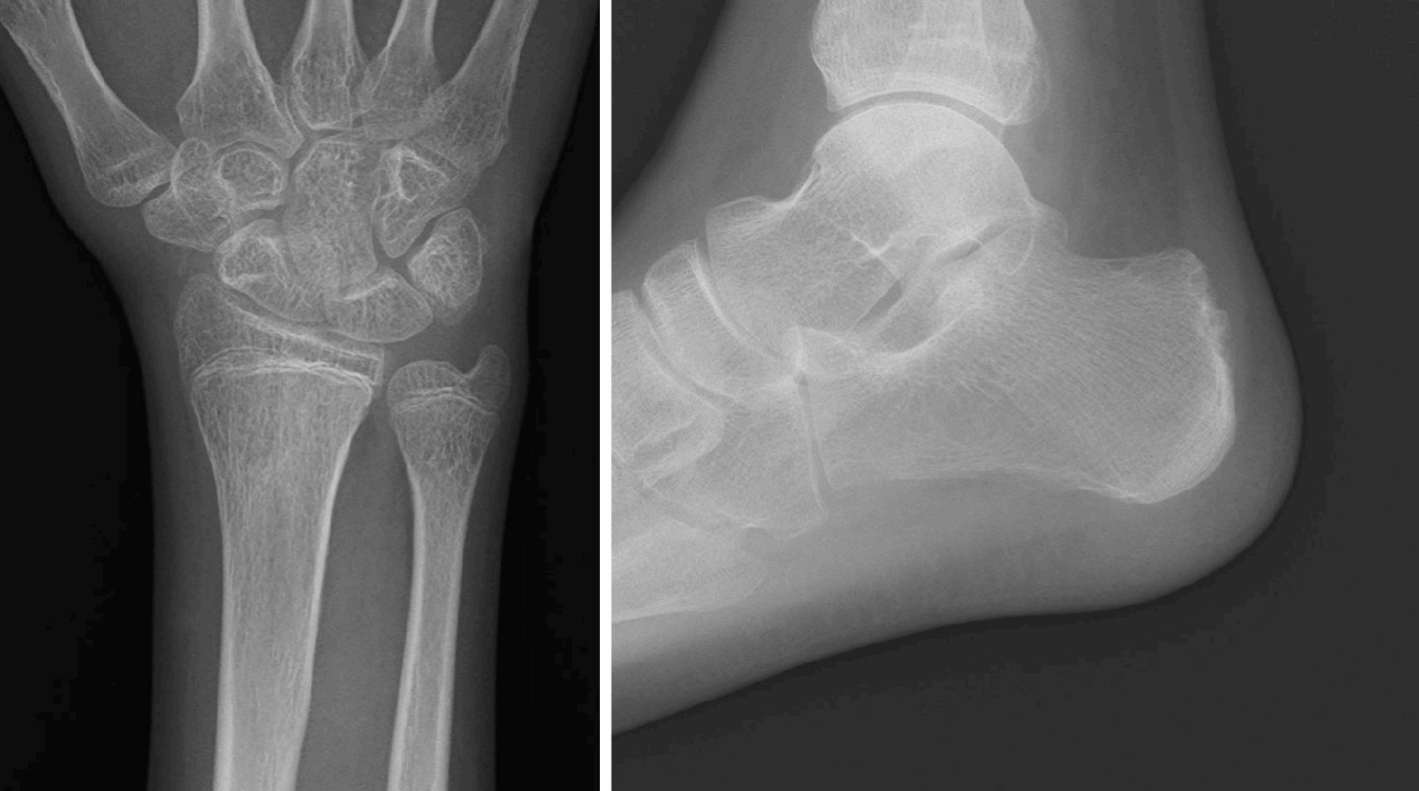
Radiographs obtained at the final follow-up, 3 years after the initial visit. No significant skeletal deformities or long-term complications were observed.

CRMO was suspected, and treatment was limited to analgesics. Symptoms gradually improved with acetaminophen, and no skeletal deformities or long-term complications were observed (Figure 4).

Bone biopsy was not performed. However, due to a lack of improvement with antibiotics, bacterial osteomyelitis was ruled out. Because the conditions were not progressive, malignancy and Langerhans cell histiocytosis were also excluded. Based on the Bristol diagnostic criteria for CRMO ^(7)^ (Table 1), a diagnosis of CRMO was made.

**Table 1.** Bristol Diagnostic Criteria for CRMO.

**The presence of typical clinical findings** (bone pain +/- localised swelling without significant local or systemic features of inflammation or infection)
**AND**
**The presence of typical radiological findings** (plain x-ray: *showing combination of lytic areas, sclerosis and new bone formation* or preferably STIR MRI: *showing bone marrow oedema +/- bone expansion, lytic areas and periosteal reaction*)
*AND EITHER*
**Criterion 1:** more than one bone (or clavicle alone) without significantly raised CRP (CRP < 30 g/L).
OR
**Criterion 2:** if unifocal disease (other than clavicle), or CRP >30 g/L, with bone biopsy showing inflammatory changes (plasma cells, osteoclasts, fibrosis or sclerosis) with no bacterial growth whilst not on antibiotic therapy.

The original paper ^(7)^ is published under the CC BY license. Therefore, no request for permission to reprint is required.

## Discussion

Previous studies have reported an association between CRMO and IBD, with CRMO preceding IBD in 53% of cases, while IBD appears first in 35% ^(6)^. This highlights the need for clinicians to consider CRMO when patients with IBD present with unexplained musculoskeletal pain. Diagnosis is challenging due to overlapping features with bacterial osteomyelitis, particularly in immunocompromised patients. MRI has a high sensitivity in detecting CRMO and can identify lesions even in asymptomatic cases, making it useful for both initial diagnosis and follow-up. In the present case, performing MRI of both wrist joints at disease onset might have enabled an earlier diagnosis ^(8)^.

Although the relationship between IBD and CRMO, or IBD and PSC, has been recognized for some time, each condition is rare, and the molecular biological relationship remains unclear. Only one prior case involving the combination of IBD, CRMO, and PSC―similar to this case―has been reported ^(5)^. In that case, CRMO was diagnosed based on nonbacterial osteomyelitis in both femurs and was followed by a diagnosis of PSC due to persistent elevation of liver enzymes. Further investigation into the association between PSC and UC in that case revealed the presence of asymptomatic UC. In contrast, in the present case, UC and PSC were already being managed with strict immunosuppressive therapy when osteomyelitis of the right distal radius developed. A key difference from the previous case is that all three conditions were symptomatic, with CRMO being the last to develop. The combination of CRMO and UC is frequently reported, and the coexistence of all three conditions may be underpinned by genetic and immunological factors that remain unclear. Vedolizumab has been shown to have an effect on UC, but there are no reports mentioning its effect on CRMO. Accumulating case reports of complicated cases like this one will help clarify the immunological and genetic differences between CRMO and UC. Unfortunately, these investigations were not performed in this case because the patient and family declined them. This is a limitation of this case.

Nonsteroidal anti-inflammatory drugs are typically the first-line therapy for CRMO. Biologic agents have shown efficacy in treating both CRMO and IBD. Alternative therapies, such as bisphosphonates or interleukin-1 inhibitors, may be considered in refractory cases ^(4)^.

In conclusion, an important point from this case report is that nonbacterial osteomyelitis occurred in a patient with IBD and PSC. This case report supports the avoidance of unnecessary antibiotic use for osteomyelitis in patients with IBD and PSC, with consideration of the possibility of CRMO.

## Article Information

### Author Contributions

Study design, conceptualization, and proof outline: Yuta Tsukagoshi,. Data collection: Yuto Nishiura and Yuta Tsukagoshi. Interpretation of results and manuscript preparation: Yuto Nishiura and Yuta Tsukagoshi. Project supervision: Yuta Tsukagoshi. Manuscript writing: Yuto Nishiura and Yuta Tsukagoshi. All authors discussed the results and commented on the manuscript.

### Conflicts of Interest

None

### IRB Approval Code and Name of the Institution

No, Ethical approval was not required for this case report, as it does not involve interventional research. Parental consent was obtained for the use of radiographs and clinical photographs included in this study.
